# A Report on the Yellow Fever That Recently Prevailed at Woodville, Miss.

**Published:** 1845-02

**Authors:** C. De Valetti, Thomas M. Logan

**Affiliations:** A committee delegated for the purpose; A committee delegated for the purpose


*A Report on the Yellow Fever that recently prevailed at Woodville, Miss. Read in French and English before the Medico-Chirurgical Society of Louisiana, on the* 2d *October*, 1844. By Doctors C. DE VALETTI and THOMAS M. LOGAN, a committee delegated for the purpose.—Before submitting to you an account of the scientific mission that we accomplished in the name of the Society, it is but rendering justice to commence with thanking our distant Confrères, who aided us in our researches, and who, regardless of their fatigue, afforded us both their time and their skill, with a readiness which cannot be too much praised.

We commend to your notice Drs. M'Kelvey and Austin, of Bayou Sara, La.; and Drs. Stone, Martin, A. C. Holt, and Kilpatrick, of Woodville, Miss.—On the 19th September, 1844, at half past 5 o'clock, P. M., we arrived at Bayou Sara. Immediately we called upon Drs. M'Kelvey and Austin, who informed us that their town was comparatively healthy, and that but one single case of Yellow Fever had occurred, which came from Woodville, where, they said, it was raging epidemically. This case was that of Mr. C. M. Stewart, a delegate of the Whig Convention from Natchez, who after having passed three days at Woodville, left there on the 4th September, and returned to his plantation, near Natchez, where he remained some days. After this he went to Bayou Sara, where he died on the 12th September, after a very short illness, with black vomit.

The Yellow Fever did not spread afterwards at Bayou Sara. This is a fact which we inquired into with all the attention it merited; for it seemed to touch very close upon the subject, which we went to try to elucidate.

The friends of Mr. Stewart, wishing to transport his remains to Mississippi, engaged a labourer at Bayou Sara to exhume the body. This man commenced his work, but as soon as he reached the coffin, was so overcome by the offensive odour, that he refused to finish his undertaking and left it.

Having returned home, he drank freely of brandy, and shortly afterwards complained of headache, from which he generally suffered whenever he drank too much. The next day being Sunday, he remained in bed. His physician, Dr. Austin, who kindly drew up for us the notes of his case, which we have copied literally, did not see him until the Tuesday morning following. He had had a violent chill all the previous night, to which fever succeeded, with the following symptoms.

The pulse, without being hard, was at 100—his face was flushed, conjunctiva injected, and he complained of his eyes burning him. The tongue was red, and his gums appeared white and somewhat swollen. Severe darting pains in the head and back distressed him, while there was considerable oppression at the præcordia and a burning pain at the epigastrium. His bowels had been constipated, and for this reason 20 grs. of blue pill mass were prescribed. On Wednesday there was no remission of the fever, nor any amelioration of the other symptoms; on the contrary, an unquenchable thirst, accompanied by exquisite tenderness in the right hypochondrium, and incessant vomiting of every thing taken into the stomach, mixed with bile, developed themselves.

Two scarified cups were now applied to the epigastrium and right hypochondrium, and immediately afterwards, a sinapised cataplasm to these parts. The first dose of blue mass having failed to produce an evacuation, 15 grains more were administered. In the evening the vomiting ceased, and a remission of the fever and most of the attendant symptoms occurred. A dose of castor oil was now prescribed, and with its operation the fever disappeared. On Thursday, sulphate of quinine in large doses was given every two hours, and on Friday the patient was discharged cured.

In spite of the similitude of the initial symptoms, it is for you, gentlemen, to decide, whether, viewing the treatment employed, in connection with the severity of all the symptoms, in particular the chill so intense as to last a whole night; viewing the length of time, which elapsed between the invasion of the disease, together with the administration of the first remedy; it is for you, we repeat, to decide, whether we were correct in regarding this as one of the common inflammatory bilious fevers of the country, aggravated by the putrid exhalations and the inebriety to which the patient was exposed, rather than a case of Yellow Fever.

At all events, both the attending, and the consulting physician, Dr. M'Kelvey, concurred with us in our opinion of the case.

Thus advised, we departed the next day for Woodville, where we arrived at half past 8 o'clock, A. M.

Without delay we reported ourselves and the object of our mission to Drs. Stone, Martin, and Holt, who forthwith conducted us to their respective patients in succession.

After visiting several cases, we experienced much difficulty in pronouncing upon the nature of the disease.—It greatly resembled Yellow Fever, but not altogether. As yet, understand, gentlemen, we had only seen patients in the 3d and 4th day of their sickness,—many of whom had been violently attacked. The symptoms of the *début* existed no longer, and those at this period differed materially from the ordinary symptoms of the Yellow Fever of New Orleans.—Among all, the tongue, the gums, and the digestive organs, on which they are dependant, remained for us mute.—Thus, for example, we saw two patients, one on the 5th, and the other on the 6th day of their attack, just beginning to turn yellow, tormented with incessant vomiting, yet without much thirst, the epigastrium sensitive only upon strong pressure, the pulse at 98, the tongue large without coating, whitish only in its centre, normal at its margins, its papillæ not developed, and the gums healthy.—One, indeed, of these two patients had the tongue of a person in health. We looked, but in vain, for the peculiar anxious *facies* of Yellow Fever.—It may be conceived now why we were at first embarrassed.

Soon, however, our doubts vanished. Three patients next offered for our inspection, two of whom were physicians.

The first, Dr. Currier, aged 45, a resident of the country for 18 years, a man justly esteemed, died before our eyes, with confirmed black vomit, carrying away with him the regrets of the whole community.

With this individual the tongue and gums were almost normal, although there was the icteric teint of the eyes and skin, together with the general group of symptoms, which constitute Yellow Fever at its fatal termination.

It was the afternoon before the night of his death, that we saw him for the first time, and we then remarked a sign, in our opinion always fatal, at whatever period of the disease: it was a difficulty of respiration, which betrayed itself by a long inspiration, immediately followed by a very short expiration.

The second case, that of Dr. Proctor, afforded us an indubitable specimen of an abundant passive hemorrhage from the gums. It was the 9th day of his disease when we saw him. There was a general intense yellow colour, a hot skin particularly on the forehead, a pulse of 122, sufficiently large to make us suspect a speedy return of the hemorrhage which had been suspended since the day before, but which did not recur. The whole extent of the intestinal canal was free from pain, while the tongue and gums remained normal, with the exception of a slight mercurial stomatitis. Although he laboured under a slight sub-delirium, still he replied correctly, but slowly to the questions we put to him. A slight diminution of the motor power in the inferior members also attended. On the next day, when we took leave of him, he was evidently improving: he recognised us instantly, and we left him under the hopes of soon hearing of his recovery.

The third case was that of an Israelite, who died with cerebral congestion on the 6th day of his sickness, without any yellow colouring of the skin during life, without black vomit, without any passive hemorrhage, and with the tongue and gums healthy; but presenting nevertheless, in the highest degree, the facies and general appearance of the disease. We were so anxious to witness the changes after death, that, arriving at his residence after the funeral procession had departed, we hurried to the burial ground, where the coffin was opened for us just before the interment, and found the skin had assumed a greenish yellow.

Bear in mind that with these three patients, as well as, indeed, with all the others, the initial symptoms were precisely the same as those we recognise in Yellow Fever.

At last we had an opportunity of seeing two cases at their *début:* they were of the inflammatory type, frank and favourable. One of these two was a negro, the other white.—The ordinary symptoms obtained even to the false membrane on the gums. It was, then truly, the Yellow Fever with which we were engaged; but modified in some of its symptoms, as we thought at the time, by the topographical position of the place, where it had thus suddenly appeared. We knew not then, that this same modification, which embarrassed us so much, existed to a certain degree with the disease the present season in our own city.

Although we entertained no doubt respecting our conclusions, still we were desirous of adding to our documents the testimony of some autopsies. But our efforts to overcome the repugnance of the inhabitants were fruitless, and we were compelled to abandon the accomplishment of this *sine quâ non* of every perfect observation.—We hasten to the history of the epidemic.

Previous to the present year, Yellow Fever never prevailed at Woodville. About 5 or 6 years ago, however, an individual, flying from the epidemic, which reigned at Bayou Sara, went to Woodville, where he soon fell sick, and died with black vomit.—Neither before nor since, until the present time, has the disease ever been heard of there.

About the 9th August, 1844, Dr. Stone was sent for to see a negro man, who had been residing in the country a number of years, and who had been taken sick at his master's plantation, about 6 miles from town. The Doctor did not suspect at first the true nature of the malady: he was only struck with the great difference of the symptoms from those of ordinary fever of the country. This negro was the coachman of the family, and in the daily habit of driving in and out the town. He was bled copiously, and purged with calomel. On the 4th day a perfect intermission occurred; indeed, he was thought to be convalescent. Anticipating, however, a relapse, which is common among the fevers of the country, Dr. S. ordered the sulphate of quinine freely for him, which produced a furious delirium, in which state the patient soon died.—This death, it will be borne in mind, happened on a plantation 6 miles from town, and it is worthy of note that no other case occurred here, with the exception of a slight fever about 3 weeks after, in another negro, who had never been near the first, and who soon recovered after a purgative of calomel, without quinine.

The next case occurred in the town on the 11th August, in the person of Mr. Simrall. This gentleman was a Kentuckian by birth, and had been residing at Woodville about 3 years. His occupation was that of a merchant, and it is not remembered that he had recently received any merchandise from New Orleans, or been absent from the town since his removal there. He was attacked with the most unequivocal symptoms of Yellow Fever, which readily yielded to the free use of the lancet and calomel. After this he was put on the quinine treatment, which disagreed much with him, and rendered his convalescence tedious.

After this case the disease began to spread as an epidemic, very generally. In a few words here are the symptoms.

The disease was ushered in more or less invariably with a chill, preceded by an indefinable *malaise* and depression of the intellectual and physical energies. The reaction soon followed with but little intensity, and especially little tenacity. The face was red, the eyes often injected, and a supra-orbital cephalalgia with rachialgia generally attended. The skin was pungent, although constantly moist, and the pulse ranged from 100 to 120. The tongue was generally almost normal, and the gums, but slightly tumified, were covered with a pseudo-membranous coating, when not under the influence of mercury. The intestinal canal rarely partook of the localization, which rather obtained in the brain, and its meninges. The disease was slow in its progress, and it was not until towards the 8th and 9th day that the grave symptoms, such as black vomit and hemorrhages, appeared. The suppression of urine was a rare occurrence—but one instance of it was related to us. The yellow colour of the eyes and skin took place also very late in most of the cases. Ecchymoses were seldom perceptible even after death, and were never seen during life.

The treatment consisted of venesection, scarified cups to the back, an epispastic to the epigastrium, to calm the vomiting, and, especially, calomel in the maximum dose, viz.: 50 or 60 grs. at first—next 15 grs. every 3 hours—afterwards 10 grs.—and finally 5 grs.

This is succinctly the plan that was generally adopted. We would add here, as a particular type of the epidemic, that all the symptoms of the first period, thus combatted, yielded readily, from the 3d to the 5th day, either to a state of perfect convalescence, or to a languid condition—a sort of incubation, if we may so term it, of the more serious symptoms, which, as we have already pointed out, did not present until the 8th or 9th day.

Let us proceed to the topography of the country.

Woodville is an inland town, situated in Wilkinson County, near the South-West corner of the State of Mississippi, Lat. 31° 10′, and 8 miles from the boundary line of Louisiana. The distance in a direct line from the Mississippi River, which is the nearest river or swamp, is 15 miles, but as the whole surrounding country is rolling, and much broken, the route by rail road or stage is about 26 miles. The town is elevated to the height of 340 feet above the bank of the Mississippi River, as has been well ascertained by the Engineers who constructed the rail road from Bayou Sara and St. Francisville, which terminates here. The town covers a space of about 8 or 10 acres; the houses are not crowded together,—the streets are wide and planted with trees, and there is a large public square in the centre. The population has increased very gradually since the first settlement of the town, about 40 years ago. No sudden immigration of any consequence has ever been observed previously to the last 3 years, during which period some Dutch emigrants have located themselves here; but the number is inconsiderable. The soil of the town and adjoining country is rather worn, and consists of a mixture of clay and sand to the depth of about 15 feet, where a thick stratum of gravel next presents, through which numerous springs of clear, wholesome, and pleasant water are occasionally found percolating.

The sand rather predominates in the bottoms and low spots, for the water which collects occasionally here and forms ponds, remains perfectly sweet and transparent until dried up.

The productions of the country are chiefly cotton and corn, and the natural growth beech, magnolia, pine, &c.

The inhabitants of Woodville and the surrounding country have always enjoyed a great degree of health, seldom interrupted except by an occasional and partial prevalence of the usual autumnal fevers, to which our whole Southern alluvial country is more or less subject.

The past summer has been uncommonly hot: the thermometer being seldom below 80°, and frequently up to 100°. The whole month of June was rainy, but since, little or no rain has fallen, and the country is suffering from the want of water. The atmosphere, generally pure and dry, was so highly charged with electricity about the beginning of September, and the lightning at one time flashed so incessantly and vividly, as to create ominous apprehensions among many.

The prevalence of the winds has been from N. N. West, and the nights have not been uncommonly cool.

Such are the appreciable conditions under the influence of which, the epidemic, whose history we have just traced, developed itself at Woodville. At the day of our departure, the 21st September, the disease had already attacked about four-fifths of the population, which, embracing the space of one mile above, and two below the town, consists of about 750 inhabitants, including negroes, the proportion of whom we have not been able to ascertain. Of this total number, 150 to 200 persons left in order to escape the pestilence. Reckoning then

43 deaths among the whites, and 20 among the blacks, we have a mortality of almost one in six.

The question, gentlemen, which has been for some time before the Society, is that of determining the origin and mode of transmission of Yellow Fever. In consequence of an unfortunate error, our efforts were this year defeated at the Custom House, and we were desirous of profiting by the occasion which offered for observing Yellow Fever at its first appearance in a circumscribed locality. We went to Woodville, hoping to extract from the disease the secret of its transmission, or at least of its origin.

Before drawing up a general summary, based upon the facts which we have had the honour of narrating to you, we would first know, if there are any doubts in your minds as to the nature of the disease, whose history we have just detailed to you. Is it indeed the yellow fever?—Lest you may be vacillating on this point, we will here add as complemental facts; *primo*, that the disease never attacked those who were acclimated to yellow fever regions; *secondo*, that negroes, *cæteris paribus*, were not attacked as frequently, and seldom so seriously as the whites.—We leave these facts, then, to answer your doubts, if any there be.

At Bayou Sara, where yellow fever often prevails epidemically, and where it has not existed thus far, this year, two patients arrived under the influence of the disease from Woodville—one of whom died in the course of 4 days and the other recovered. Still Bayou Sara continues free from the disease.

At Woodville the epidemic appeared without the influence of any foreign transmission, *as far as our informations extends.* It is so small a place, that we presume if the disease had been imported, we should have heard of it. The first authentic case occurred in the negro, as reported by Dr. Stone, who was in the daily habit of driving into the town, and died at his master's plantation without transmitting his disease to any one.

Here also we would note two other important particulars respecting the mode of transmission. Beyond one mile above and two miles below the town, which form the circumscribed limits of the population, no one has been attacked, who has not overstepped this boundary and entered into the town.

One hour's stay in the town was sufficient to cause the disease in any one who was not acclimated to yellow fever.

Add to these, the circumstance already mentioned, that 5 or 6 years ago an individual left Bayou Sara then infected, and died at Woodville with black vomit, and with his death the disease died too.

Here are all the facts we have yet been able to collect: it is, gentlemen, for you to choose between the system of contagion and that of infection. It is for you we went to these places—you then must be the arbitrators.

We would, in conclusion, remark, that we have not said one single word on the etiology of the disease. Had it been necessary for us to abandon our occupations, in order to run after the research of its causes, we never would have left here, convinced that in its present state science is on this point impotent. We want no other proof of this assertion, than this very epidemic at Woodville, which developed itself under conditions altogether opposed to those of the countries where yellow fever usually reigns.

NOTE.—Since the above was handed us for publication, Dr. Logan has received, in his official capacity as corresponding secretary of the Med. Chir. Socy. of La., a communication from Dr. M'Kelvey of Bayou Sara, stating that the hack-driver, who drove Drs. De Valetti and Logan to Woodville, where he remained the part of 2 days and a night, was taken sick 3 days after his return to Bayou Sara with unequivocal symptoms of yellow fever. He convalesced on the 6th day, although there was hemorrhage from the gums. In this communication Dr. M'Kelvey says that Bayou Sara still continues free from yellow fever, although fevers of an intermittent type, attended with much pain in the head and lumbar region, and occasionally considerable inflammation of the conjunctiva prevail there. The latter symptom he attributes to the prevalence of an unpleasant raw N. E. wind, superadded to the continued dry dusty weather, and not to any affinity of the disease with the epidemic of Woodville.—(EDITORS.)

*New Orleans Medical Journal.*

